# Effect of Mixed Reality on Delivery of Emergency Medical Care in a Simulated Environment: A Pilot Randomized Crossover Trial

**DOI:** 10.1001/jamanetworkopen.2023.30338

**Published:** 2023-08-28

**Authors:** Jason Lawson, Guy Martin, Payal Guha, Matthew Gold, Amr Nimer, Sadie Syed, James Kinross

**Affiliations:** 1 Department of Surgery and Cancer, Imperial College London, London, United Kingdom; 2 Imperial College Healthcare NHS Trust, London, United Kingdom

## Abstract

**Importance:**

Mixed-reality (MR) technology has the potential to enhance care delivery, but there remains a paucity of evidence for its efficacy and feasibility.

**Objective:**

To assess the efficacy and feasibility of MR technology to enhance emergency care delivery in a simulated environment.

**Design, Setting, and Participants:**

This pilot randomized crossover trial was conducted from September to November 2021 at a single center in a high-fidelity simulated environment with participants block randomized to standard care (SC) or MR-supported care (MR-SC) groups. Participants were 22 resident-grade physicians working in acute medical and surgical specialties prospectively recruited from a single UK Academic Health Sciences Centre. Data were analyzed from September to December 2022.

**Intervention:**

Participants resuscitated a simulated patient who was acutely unwell, including undertaking invasive procedures. Participants completed 2 scenarios and were randomly assigned to SC or MR-SC for the first scenario prior to crossover. The HoloLens 2 MR device provided interactive holographic content and bidirectional audiovisual communication with senior physicians in the MR-SC group.

**Main Outcomes and Measures:**

The primary outcome was error rate assessed via the Imperial College Error Capture (ICECAP) multidimensional error-capture tool. Secondary outcomes included teamwork (Observational Teamwork Assessment for Surgery [OTAS]; range, 0-6 and Teamwork Skills Assessment for Ward Care [T-SAW-C]; range, 1-5), scenario completion, stress and cognitive load (NASA Task Load Index [NASA-TLX; range 0-100]), and MR device user acceptability.

**Results:**

A total of 22 physicians (15 males [68.2%]; median [range] age, 28 [25-34] years) were recruited. MR technology significantly reduced the mean (SD) number of errors per scenario compared with SC (5.16 [3.34] vs 8.30 [3.09] errors; *P* = .003), with substantial reductions in procedural (0.79 [0.75] vs 1.52 [1.20] errors; *P* = .02), technical (1.95 [1.40] vs 3.65 [2.03] errors; *P* = .01), and safety (0.37 [0.96] vs 0.96 [0.85] errors; *P* = .04) domains. MR resulted in significantly greater scenario completion rates vs SC (22 scenarios [100%] vs 14 scenarios [63.6%]; *P* = .003). It also led to significant improvements in the overall quality of teamwork and interactions vs SC as measured by mean (SD) OTAS (25.41 [6.30] vs 16.33 [5.49]; *P* < .001) and T-SAW-C (27.35 [6.89] vs 18.37 [6.09]; *P* < .001) scores. As reported via mean (range) NASA-TLX score, there were significant reductions for MR-SC vs SC in participant temporal demands (38 [20-50] vs 46 [30-70]; *P* = .03) and significant improvements in self-reported task performance (50 [30-60] vs 39 [10-70]; *P* = .01). Overall, 19 participants (86.4%) reported that they were more confident in making clinical decisions and undertaking clinical procedures with MR support.

**Conclusions and Relevance:**

This study found that the use of MR technology reduced error, improved teamwork, and enhanced practitioner confidence when used to support the delivery of simulated emergency medical care.

**Trial Registration:**

ClinicalTrials.gov Identifier: NCT05870137

## Key Points

**Question:**

Can mixed reality play a role in providing a safe and feasible adjunct to enhance emergency medical care in a simulated environment?

**Findings:**

This pilot randomized crossover trial in 22 physicians found that mixed reality led to a reduced error rate in emergency clinical care scenarios compared with a standard care intervention. The most substantial reductions in error were represented in procedural, technical, and safety domains.

**Meaning:**

These findings suggest that mixed-reality technology may have a role to play in reducing variance and improving the quality of care in distributed clinical environments.

## Teaser

This randomized crossover trial investigates the effect of mixed-reality technology on error rate among physicians in emergency clinical care scenarios.

## Introduction

Delivering care and leveraging access to remote specialists through new technologies, such as mixed reality (MR), has become a favored approach to optimizing clinical care and patient outcomes, as well as widening accessibility and reducing cost.^1,2,3,4,5^ MR technology has been used across a range of clinical scenarios, including perioperative planning, surgical training, and 3D telemedicine support,^6,7,8^ and deployed to support the delivery of clinical care during COVID-19.^9^ With recent evidence suggesting an array of promising use cases for the technology,^10,11,12,13^ there is increasing potential for MR to augment the delivery of emergency medical care by delivering clinically relevant holographic content and live communication across geographically disparate and remote environments.^14^ In addition to its role in augmenting and enhancing direct clinical care, the technology has the potential to provide a means to capture, store, and annotate data obtained during routine clinical interactions that may populate digital data repositories to support future clinical care, education, and research. It may also lead to better quality assurance and governance through novel performance and outcome insights.^15^ Despite MR’s potential, the technology remains relatively immature and there are important questions concerting its sustainability, scalability, and cost-effectiveness.

To our knowledge, no randomized trials of MR technologies have been performed to assess their impact vs standardized methods of education and training for resuscitation scenarios or direct clinical care in the acute setting. The aim of this prospective trial was therefore to investigate whether an MR platform could enhance the delivery of emergency clinical care by reducing error and improving individual and team performance in a simulated environment.

## Methods

### Study Design

Full prospective ethical approval and study registration were obtained for this randomized crossover trial, and all participants provided written informed consent. The study was conducted and reported according to the Consolidated Standards of Reporting Trials (CONSORT) reporting guideline (eTable 1 in Supplement 1), with the full prospectively approved trial protocol and statistical analysis plan provided in Supplement 2.

Resident-grade physicians working in acute medical and surgical specialties were prospectively identified and invited to participate through the medical education department of a single UK tertiary Academic Health Sciences Centre. The study was conducted from September to November 2021. Participants received a standardized MR platform headset and operational tutorial and completed a qualitative questionnaire to determine their clinical experience and exposure to the technology prior to commencing the study. The facilitated tutorial provided a practical introduction to the fundamentals of use, how to make and receive calls, and how to use clinical data assets. A competency test permitted progress to the trial phase, ensuring standardized basic technical competency and mitigating device-related first-use learning effects.

**Figure 1.**
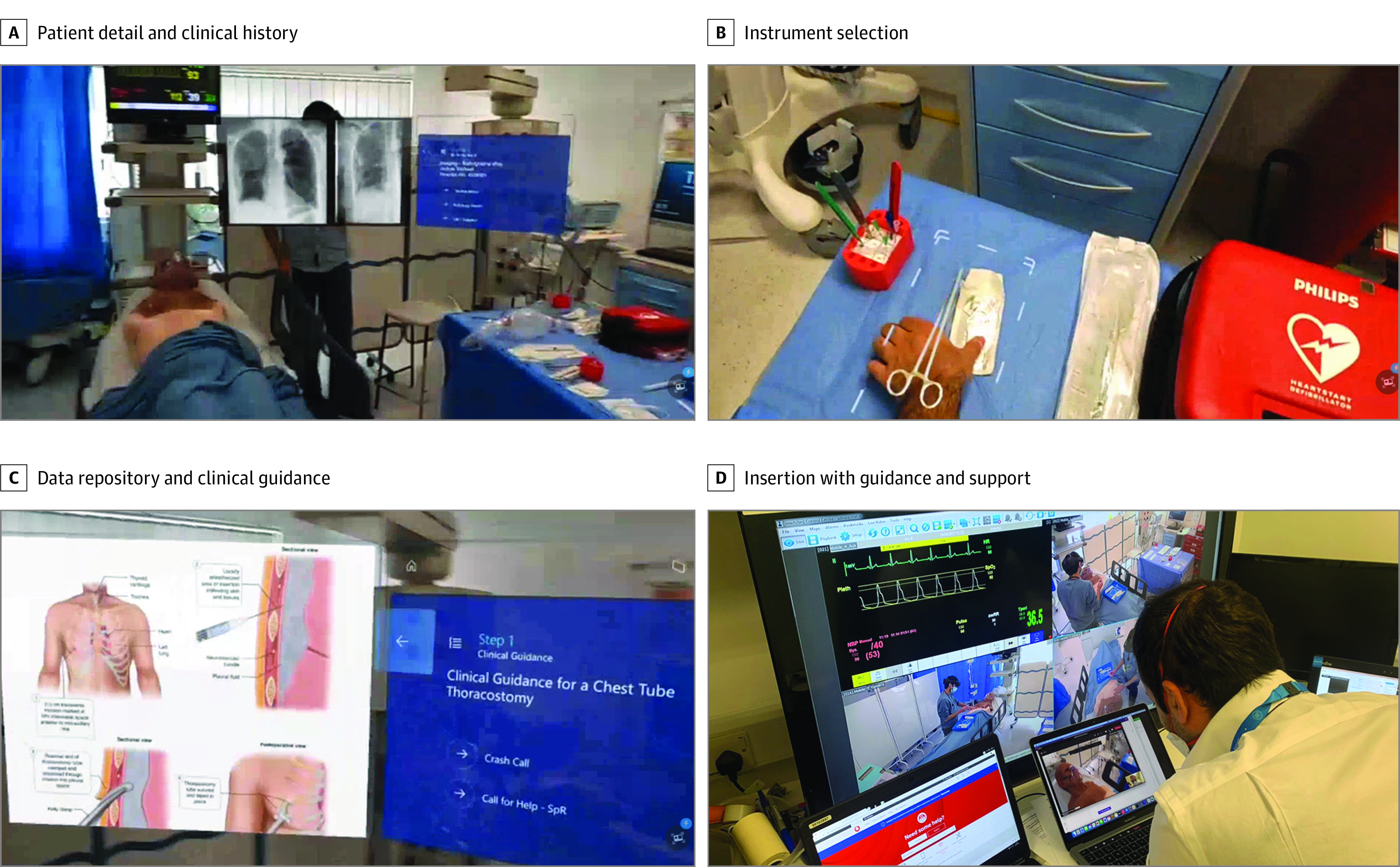
Use of Mixed-Reality Device A, Patient detail and clinical history are provided bedside, representing true head-up display functionality with the ability to place 2D and 3D objects within a user’s visual field. B, Emergency thoracotomy instrument selection is facilitated through inclusion of mixed-reality assets and via live, bidirectional communication with senior support via video and voice. C, Demonstration of the digital data repository and clinical guidance use. D, Insertion of chest drain with guidance and senior support through remote assistance and mixed-reality device support, representing safety areas for chest drain insertion.

Participants completed 2 simulated clinical resuscitation scenarios and were randomized via block randomization to standard care (SC) or MR-supported care (MR-SC) groups for their first scenario prior to crossover and analysis by care group to account for learning and carryover effects. Clinical scenarios were based on standardized clinical moulages undertaken for advanced life support qualifications and those undertaken by physicians in training as part of their core curriculum. SC was delivered by participants with access to didactic instruction, standard written clinical support tools, and telephonic support. Resources inclusive of radiology, blood results, patient notes, and clinical guidelines were available through standard ward-base computer systems. Access to senior support was provided through telephones and a bleep or on-call system standard to practice within the host institution. The MR-SC scenario made these resources available through the MR device. Participants were able to access patient data, visualize clinical guidance, and access interactive 3D content, including patient-specific visualization and guidance to aid with clinical procedures, in addition to bidirectional audiovideo calls to senior support (Figure 1).

The study was performed at a specialist high-fidelity simulation suite located within a UK Academic Health Sciences Centre. Sessions were facilitated and led by experienced simulation practitioners (M.G. and A.N.). A high-fidelity adult patient simulator (Laerdal Medical Ltd) was used to simulate an adult who was unwell and required resuscitation, including procedural intervention in the form of a chest drain insertion. Clinical scenarios are provided in the eTable in Supplement 1. All scenarios were video recorded, assessed, and rated by 2 independent researchers (J.L. and G.M.) who were blinded to participant randomization and the order in which the scenario took place.

The primary outcome measure was error rate as assessed by the Imperial College Error Capture (ICECAP) tool, a validated, multidimensional assessment of error.^16^ This tool assesses error across 6 primary domains, including equipment, communication, procedure-independent pressures, and technical, safety, and patient-related factors, with a further domain for other to capture domains not specified.^16^ The tool defines an error as an event that initially fails to achieve its intended outcome or prevents clinical scenarios from proceeding in an ideal manner. These may include wrong acts, omissions, inefficiencies, delays, safety failures, and problems that cause harm or delay care; these acts may cause harm or have the potential to cause harm.

Further exploratory analyses were undertaken, including of time to complete the clinical scenario and global assessments of team performance via 2 specific assessment tools. First, the Observational Teamwork Assessment for Surgery (OTAS) tool^17^ was used. This tool captures a comprehensive assessment of the quality of teamworking and team interactions across 5 domains on a scale of 0 to 6: communication, coordination, cooperation, leadership, and situational awareness. Second, the Teamwork Skills Assessment for Ward Care (T-SAW-C) tool^18^ was used. This tool assesses 6 domains (decision-making, situational awareness, cooperation, coordination, and communication) on a scale of 1 to 5. Workload was assessed by asking participants to complete the NASA Task Load Index (NASA-TLX) score.^19^ The NASA-TLX is a subjective, multidimensional assessment tool that rates perceived workload to assess task, system, or team effectiveness or other aspects of performance using a Likert score in 6 domains (score range, 0-100). Qualitative feedback and user experience data were collected via a standardized questionnaire using free text and Likert scale responses after completion of the study (eAppendix in Supplement 1).

### MR Technology

The MR device used was HoloLens 2 (Microsoft), an untethered, head-mounted device that permits users to view 3D holographic images as part of their surrounding environment.^20^ Figure 1A to C demonstrates this holographic content placed in the visual field of the participant within the simulated environment of the trial. Remote Assist is part of the Microsoft Dynamics 365 suite and facilitates remote collaboration and support, as demonstrated in Figure 1D. Locally developed software was used to provide additional functionality beyond the limitations of Remote Assist; as a stand-alone application, Remote Assist cannot integrate remote participant views and 2D annotations with other bespoke MR assets. The software enabled full 3D annotation and the ability to share the state of MR assets between participants.

### Statistical Analysis

Standard descriptive statistics were used. The distribution of data was checked for normality using the Shapiro-Wilks test. Comparison of scores between groups was made by paired *t* test for parametric data and a Mann-Whitney *U* test for nonparametric data. Statistical significance was set at *P* ≤ 0.05, and statistical tests were 2-sided. Analysis was performed on GraphPad Prism version 9 (GraphPad Software). Data were analyzed from September to December 2022.

## Results

A total of 22 resident-grade physicians (15 males [68.2%]; median [range] age, 28 [25-34] years) were prospectively recruited. All participants had prior experience or qualifications in advanced life support skills and resuscitation. There were 2 participants (9.1%) with 1 year of clinical practice since their primary medical qualification, 14 participants (63.6%) with at least 2 years, and 6 participants (27.3%) with 3 years or more. There were 2 participants (9.1%) who had previously used the MR device analyzed in this study, 18 participants (81.8%) who had heard of the technology but not previously used a device, and 2 participants (9.1%) had never heard of or used the technology. All participants were able to safely use the device without impediment, and there were no technical failures of wireless connectivity or data recording during the trial.

### Primary Outcome: Error Rate

**Figure 2.**
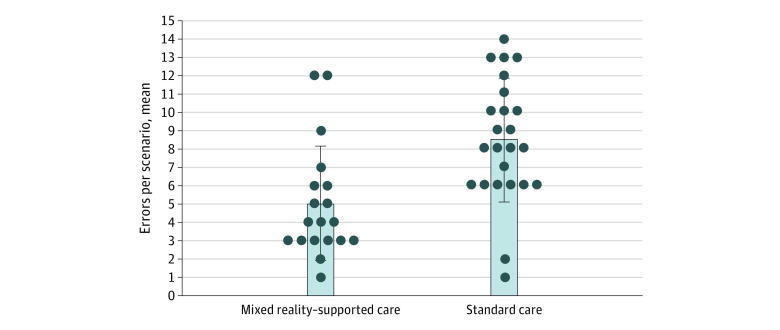
Overall Errors for Mixed Reality vs Standard Care The overall mean number of errors recorded per scenario are presented for mixed reality–supported care and standard care (5.16 vs 8.30 errors; *P* = .003). Dots indicate individual participant scores; whiskers, IQRs.

Overall, the use of the MR device to enhance standard care resulted in significantly fewer mean (SD) errors per scenario vs SC (5.16 [3.34] vs 8.30 [3.09] errors; *P* = .003) (Figure 2). Significantly fewer mean (SD) errors per scenario were seen in procedural (0.79 [0.75] vs 1.52 [1.20] errors; *P* = .02), technical (1.95 [1.40] vs 3.65 [2.03] errors; *P* = .01), and safety (0.37 [0.96] vs 0.96 [0.85] errors; *P* = .04) domains. There was no significant reduction in communication errors or equipment errors.

### Secondary Outcomes: Task Completion and Time

Participants took significantly longer to complete the MR-SC scenario compared with the SC scenario (mean [SD] time, 11.12 [4.27] minutes vs 8.57 [5.27] minutes; *P* = .03). However, there was significant improvement in task completion rates for MR-SC vs SC (22 scenarios [100%] vs 14 scenarios [63.6%]; *P* = .003).

### Secondary Outcomes: Analysis of Individual and Team Performance

The use of the MR platform to enhance standard care led to significant improvements in overall quality of teamwork and interactions, as demonstrated by mean (SD) overall scores for MR-SC vs SC in OTAS (25.41 [6.30] vs 16.33 [5.49]; *P* < .001) and T-SAW-C (27.35 [6.89] vs 18.37 [6.09]; *P* < .001) assessment. Scores across individual domains of OTAS and T-SAW-C assessments are represented in Table 1.

**Table 1.** Team Performance Scores Per Scenario

Domain	Score, mean (SD)		*P* value
	SC	MR-SC	
**OTAS**			
Overall	16.33 (5.49)	25.41 (6.30)	<.001
Behavior	2.78 (1.17)	4.26 (1.33)	.005
Communication	3.22 (0.95)	4.37 (1.12)	.002
Coordination	2.78 (0.90)	4.36 (1.28)	.001
Cooperation	2.65 (1.11)	4.26 (1.28)	.001
Leadership	2.17 (1.19)	3.89 (1.37)	.001
Monitoring	2.83 (1.07)	3.95 (1.27)	.004
**T-SAW-C**			
Overall	18.37 (6.09)	27.35 (6.89)	<.001
Behavior	2.65 (1.07)	3.84 (1.21)	.01
Communication	3.22 (0.80)	4.05 (0.78)	.007
Coordination	2.74 (0.86)	4.00 (1.05)	.002
Cooperation	2.61 (0.99)	4.16 (1.01)	<.001
Leadership	2.13 (1.14)	3.63 (1.16)	<.001
Monitoring	2.78 (1.04)	3.63 (1.16)	.009
Decision-making	2.26 (1.32)	4.00 (1.15)	.001

Abbreviations: MR-SC, mixed reality–supported care; OTAS, Observational Teamwork Assessment for Surgery; SC, standard care; T-SAW-C, Teamwork Skills Assessment for Ward Care.

### Secondary Outcomes: Task Load and User Experience

In analysis of the NASA-TLX, temporal demand (perceived time pressure) was significantly reduced by wearing the MR device vs SC (mean [range] score, 38 [20-50] vs 46 [30-70]; *P* = .03). Self-reported task performance was also significantly improved when using the MR device vs SC (mean [range] score, 50 [30-60] vs 39 [10-70]; *P* = .01). All other domains should no significant difference between groups (Table 2).

**Table 2.** Workload Scores by Domain

Domain	NASA-TLX score, mean (range)		*P* value
	SC	MR-SC	
Mental demand	44 (20-60)	44 (30-60)	>.99
Physical demand	26 (10-40)	34 (10-70)	.08
Temporal demand	46 (30-70)	38 (20-50)	.03
Effort	47 (30-60)	43 (30-70)	.22
Frustration	33 (10-70)	31 (10-70)	.74
Performance	50 (30-60)	39 (10-70)	.01

Abbreviations: MR-SC, mixed reality–supported care; NASA-TLX, NASA Task Load Index; SC, standard care.

### Qualitative Assessment and Participant Feedback

In participant feedback, 1 individual said, “The communication with a senior [clinician] during the task was helpful and useful. This is the major added value of the device. In the pressure of a medical emergency, I did not find reading a textbook [clinical guidance] diagram useful and I didn’t think the holograms to select the tools were useful. In fact, I found them confusing. I was confident doing chest drains already, though. I can imagine if you have never done a chest drain before, this side of things could be useful.” Overall, 19 participants (86.4%) agreed or strongly agreed that the MR device was comfortable to wear, and 17 participants (77.3%) agreed or strongly agreed that it was easy to interact with and control the platform. There were 20 participants (90.1%) who thought the quality of virtual objects was good and 22 participants (100%) who could see the benefit of using the technology for delivering clinical care; 13 participants (59.1%) thought that most people would learn to use this system very quickly. There was a consensus that the MR technology improved the experience of delivering clinical care; 19 participants (86.4%) reported that they strongly agreed that they were more confident in making clinical decisions, and the same number reported that they were more confident in undertaking clinical procedures. There were more modest demonstrations of how these gains translated into the perceptions of the care delivered; 15 participants (68.2%) agreed or strongly agreed that they made better decisions, performed clinical procedures to a higher quality, and made fewer errors. There were 7 participants (31.8%) who agreed or strongly agreed that the MR device provided better situational awareness; 20 participants (90.1%) who agreed or strongly agreed that the device improved communication, access to senior support, and their perception of clinical support; and 14 participants (63.6%) who agreed or strongly agreed that the device improved teamwork. A participant said, “Communication was quick, and encouragement and guidance were present remotely, which resulted in [the] correction of approach or advice on instrument use. [This] provided the user with confidence when undertaking [the] procedure.”

However, some participants reported mild adverse effects of the MR system during the simulation; 2 participants (9.1%) reported discomfort from wearing the device, and the same number reported sore eyes or a headache. Despite some anticipated adverse effects and user challenges, no participants reported that the technology impeded their ability to deliver safe care and 20 participants (90.1%) agreed or strongly agreed that they would be happy to use the device in clinical practice.

## Discussion

This single-center, prospective randomized crossover trial found that wearable MR technology improved the performance of clinicians in a simulated environment by reducing error. Specifically, the MR device improved scenario completion rates (100% vs 63.6%; *P* = .003) and reduced the overall error mean rate per scenario (5.16 vs 8.30 errors; *P* = .003).

The way in which this technology enhances performance is nuanced and may be further understood by closer examination of specific domains represented by the ICECAP tool. Equipment errors relate to availability, failure or fault of the device, its configuration, participant familiarity with the device, and device selection. Given that the equipment provided was standardized and specific to the interventions that participants were required to perform, there were understandably no significant differences seen between groups within this domain. Communication errors occur when communication has been misleading, absent, not heard, or misheard or if there is significant discord among team members. No significant differences between groups in the number of communication errors captured were seen, although findings from OTAS and T-SAW-C assessments suggested the converse. Additionally, the MR device improved communication among team members. These findings suggest that the MR device may have improved the overall quality of communication among team members but did not reduce the frequency of specific communication errors.

However, significant reductions in mean errors per scenario were seen in procedural (0.79 vs 1.52 errors; *P* = .02), technical (1.95 vs 3.65 errors; *P* = .01), and safety (0.37 vs 0.96 errors; *P* = .04) domains. Procedural and technical domains consider the psychomotor capabilities of the participant, minimizing the effect of the unfamiliarity of the procedure, improving accuracy of equipment selection and ultimately procedural technique. The ability of the MR platform to reduce errors in these 2 domains is an important finding. It highlights the potential role of the device functionality, specifically that of bidirectional video and voice communication, and procedural-specific holographic content in augmenting the ability of clinicians to perform unfamiliar procedures.

There were significantly reduced clinical error rates per scenario in the MR-SC group compared with the SC group while simultaneously significantly increasing successful task-completion rates. Improvements in clinical success and reduced clinical safety errors came at the expense of extended completion times, however, when using the MR device. This variation in completion rates and the extended time between MR-SC and SC groups may be accounted for by the number of scenarios that were stopped early in the SC group when participants were unable or unwilling to progress further with the clinical scenario due to inexperience or unfamiliarity with the procedure. Implications of these observations are important for the training and support of front-line clinicians who may require real-time support when learning to perform complex or new tasks. In addition, there is a need for clinicians to feel sufficiently confident in the support that any new technology provides and to perceive that it aids them in safely and competently performing an unfamiliar procedure.

Use of the MR platform led to significant improvements in the overall quality of teamwork and interactions for MR-SC vs SC as measured OTAS and T-SAW-C assessment tools. Interestingly, this did not occur because of improvements in the quality of communication, suggesting that holographic computer vision may make a significant contribution to improved team performance. This is likely a product of a reduced cognitive load, as evidenced by improvements in temporal demand and self-reported task performance and reports from participants that they were more confident in making clinical decisions and undertaking procedures.

Future MR systems will need to be further adapted for clinical performance. Although there were no reported adverse effects of dizziness or headaches from MR use, some participants reported that prolonged wearing of this headset caused some discomfort or sore eyes. Based on our findings, future iterations of the technology may therefore benefit from improvements in comfort and fit. Integration of MR systems with hospital information and communication technology networks for the real-time visualization of patient clinical data creates technical and data governance challenges that will need to be overcome. Ultimately, however, this analysis suggested that care augmented by MR technology may be superior to standard clinical practice for technical performance and safety, workload, and team performance metrics. Future work should now aim at delivering the seamless integration of the technology into existing systems and processes while simultaneously identifying barriers to its scalability across a range of settings and practice areas.

### Limitations

There are some limitations to this work. First, recruitment was limited due to COVID-19, and a pragmatic decision was taken to halt the trial early, restricting recruitment and participation. Second, this is a simulated trial, and it is possible that some observed benefits may be less significant when deployed in clinical environments. Furthermore, given that this was a pilot study, a number of exploratory secondary analyses were undertaken that may have introduced the risk of type I error. Third, the inability to fully blind observers to the intervention group due to the nature of the simulation and technology being trialed may have also introduced bias. Fourth, it is possible that this study was vulnerable to selection bias given that participants who offered to take part may have been more willing to adopt this novel technology. Fifth, a further key limitation to this study is the technology itself; it is relatively immature, with much scope for iterative development and improvement. It is expensive, with a requirement for expert technical knowledge to effectively create and deliver impactful and relevant content, which may prevent its adoption in resource- or knowledge-poor settings. Sixth, as with any technology-based trial, there is the potential for technology bias to influence results and for cost, resource, and technological barriers to limit adoption, scalability, and wider impact.

## Conclusions

This randomized crossover trial found that wearable MR technology improved technical performance and reduced error in clinicians performing complex tasks in high-intensity simulated environments. Use of MR most notably impacted the ability to complete complex procedural tasks, such as intercostal drain insertion for novice trainees. While MR is in its infancy, our findings suggest promise for the technology in improving the quality of care, enhancing access to specialist care in remote settings, and supporting junior medical staff to be more confident in delivering high-quality care. Not all advances that show benefits in simulated environments translate to clinical settings, so the potential benefits of this technology must now be investigated in live clinical settings in order to demonstrate its safety, effectiveness, and scalability.

## Notes

Supplement 1. **eFigure. **Flowchart **eTable.** Clinical Scenarios **eAppendix.** Questionnaire

Supplement 2.
Trial Protocol and Statistical Analysis Plan

Supplement 3.
Data Sharing Statement

## References

[1] Hollander JE, Carr BG. Virtually perfect: telemedicine for COVID-19. N Engl J Med. 2020;382(18):1679-1681. doi:10.1056/NEJMp2003539 32160451

[2] Turer RW, Jones I, Rosenbloom ST, Slovis C, Ward MJ. Electronic personal protective equipment: a strategy to protect emergency department providers in the age of COVID-19. J Am Med Inform Assoc. 2020;27(6):967-971. doi:10.1093/jamia/ocaa048 32240303 PMC7184500

[3] Rohrbach N, Gulde P, Armstrong AR, . An augmented reality approach for ADL support in Alzheimer's disease: a crossover trial. J Neuroeng Rehabil. 2019;16(1):66. doi:10.1186/s12984-019-0530-z 31159816 PMC6547460

[4] Babatunde AO, Abdulazeez AO, Adeyemo EA, Uche-Orji CI, Saliyu AA. Telemedicine in low and middle income countries: closing or widening the health inequalities gap. Eur J Environ Public Health. 2021;5(2):em0075. doi:10.21601/ejeph/10777

[5] Barbosa W, Zhou K, Waddell E, Myers T, Dorsey ER. Improving access to care: telemedicine across medical domains. Annu Rev Public Health. 2021;42(1):463-481. doi:10.1146/annurev-publhealth-090519-093711 33798406

[6] Zuo Y, Jiang T, Dou J, . A Novel evaluation model for a mixed-reality surgical navigation system: where Microsoft HoloLens meets the operating room. Surg Innov. 2020;27(2):193-202. doi:10.1177/1553350619893236 31920155

[7] Pratt P, Ives M, Lawton G, . Through the HoloLens™ looking glass: augmented reality for extremity reconstruction surgery using 3D vascular models with perforating vessels. Eur Radiol Exp. 2018;2(1):2. doi:10.1186/s41747-017-0033-2 29708204 PMC5909360

[8] Tepper OM, Rudy HL, Lefkowitz A, . Mixed reality with HoloLens: where virtual reality meets augmented reality in the operating room. Plast Reconstr Surg. 2017;140(5):1066-1070. doi:10.1097/PRS.0000000000003802 29068946

[9] Martin G, Koizia L, Kooner A, ; PanSurg Collaborative. Use of the HoloLens2 mixed reality headset for protecting health care workers during the COVID-19 pandemic: prospective, observational evaluation. J Med Internet Res. 2020;22(8):e21486. doi:10.2196/21486 32730222 PMC7431236

[10] Bala L, Kinross J, Martin G, . A remote access mixed reality teaching ward round. Clin Teach. 2021;18(4):386-390. doi:10.1111/tct.13338 33786988

[11] Minty I, Lawson J, Guha P, . The use of mixed reality technology for the objective assessment of clinical skills: a validation study. BMC Med Educ. 2022;22(1):639. doi:10.1186/s12909-022-03701-3 35999532 PMC9395785

[12] Lee Y, Kim S-K, Yoon H, Choi J, Kim H, Go Y. Integration of extended reality and a high-fidelity simulator in team-based simulations for emergency scenarios. Electronics. 2021;10(17):2170. doi:10.3390/electronics10172170

[13] García-Sevilla M, Moreta-Martinez R, García-Mato D, . Augmented reality as a tool to guide psi placement in pelvic tumor resections. Sensors (Basel). 2021;21(23):7824. doi:10.3390/s21237824 34883825 PMC8659846

[14] Ong T, Wilczewski H, Paige SR, Soni H, Welch BM, Bunnell BE. Extended reality for enhanced telehealth during and beyond COVID-19: viewpoint. JMIR Serious Games. 2021;9(3):e26520. doi:10.2196/26520 34227992 PMC8315161

[15] Tudor Car L, Kyaw BM, Teo A, . Outcomes, measurement instruments, and their validity evidence in randomized controlled trials on virtual, augmented, and mixed reality in undergraduate medical education: systematic mapping review. JMIR Serious Games. 2022;10(2):e29594. doi:10.2196/29594 35416789 PMC9047880

[16] Mason SL, Kuruvilla S, Riga CV, . Design and validation of an error capture tool for quality evaluation in the vascular and endovascular surgical theatre. Eur J Vasc Endovasc Surg. 2013;45(3):248-254. doi:10.1016/j.ejvs.2012.11.028 23305790

[17] Hull L, Arora S, Kassab E, Kneebone R, Sevdalis N. Observational teamwork assessment for surgery: content validation and tool refinement. J Am Coll Surg. 2011;212(2):234-243.e2435. doi:10.1016/j.jamcollsurg.2010.11.001 21276535

[18] Hull L, Birnbach D, Arora S, Fitzpatrick M, Sevdalis N. Improving surgical ward care: development and psychometric properties of a global assessment toolkit. Ann Surg. 2014;259(5):904-909. doi:10.1097/SLA.0000000000000451 24722223

[19] Hart SG. Nasa-Task Load Index (NASA-TLX); 20 Years later. Proc Hum Factors Ergon Soc Annu Meet. 2006;50(9):904-908. doi:10.1177/154193120605000909

[20] Pose-Díez-de-la-Lastra A, Moreta-Martinez R, García-Sevilla M, . HoloLens 1 vs. HoloLens 2: improvements in the new model for orthopedic oncological interventions. Sensors (Basel). 2022;22(13):4915. doi:10.3390/s22134915 35808407 PMC9269857

