# Supplementary material for: Effect of Mixed Reality on Delivery of Emergency Medical Care in a Simulated Environment: A Pilot Randomized Crossover Trial
Source: JAMA Netw Open. 2023 Aug 28;6(8):e2330338. doi: 10.1001/jamanetworkopen.2023.30338 (PMC10463095; doi:10.1001/jamanetworkopen.2023.30338)
Supplement: Supplement 1. — eFigure. Flowchart eTable. Clinical Scenarios eAppendix. Questionnaire [file jamanetwopen-e2330338-s001.pdf]

Supplemental Online Content

Lawson J, Martin G, Guha P, et al. Effect of mixed reality on delivery of emergency medical care in a simulated environment: a pilot randomized crossover trial. *JAMA Netw Open*. 2023;6(8):e2330338. doi:10.1001/jamanetworkopen.2023.30338

eFigure. Flowchart
eTable. Clinical Scenarios
eAppendix. Questionnaire

This supplemental material has been provided by the authors to give readers additional information about their work.

© 2023 Lawson J et al. *JAMA Network Open.*

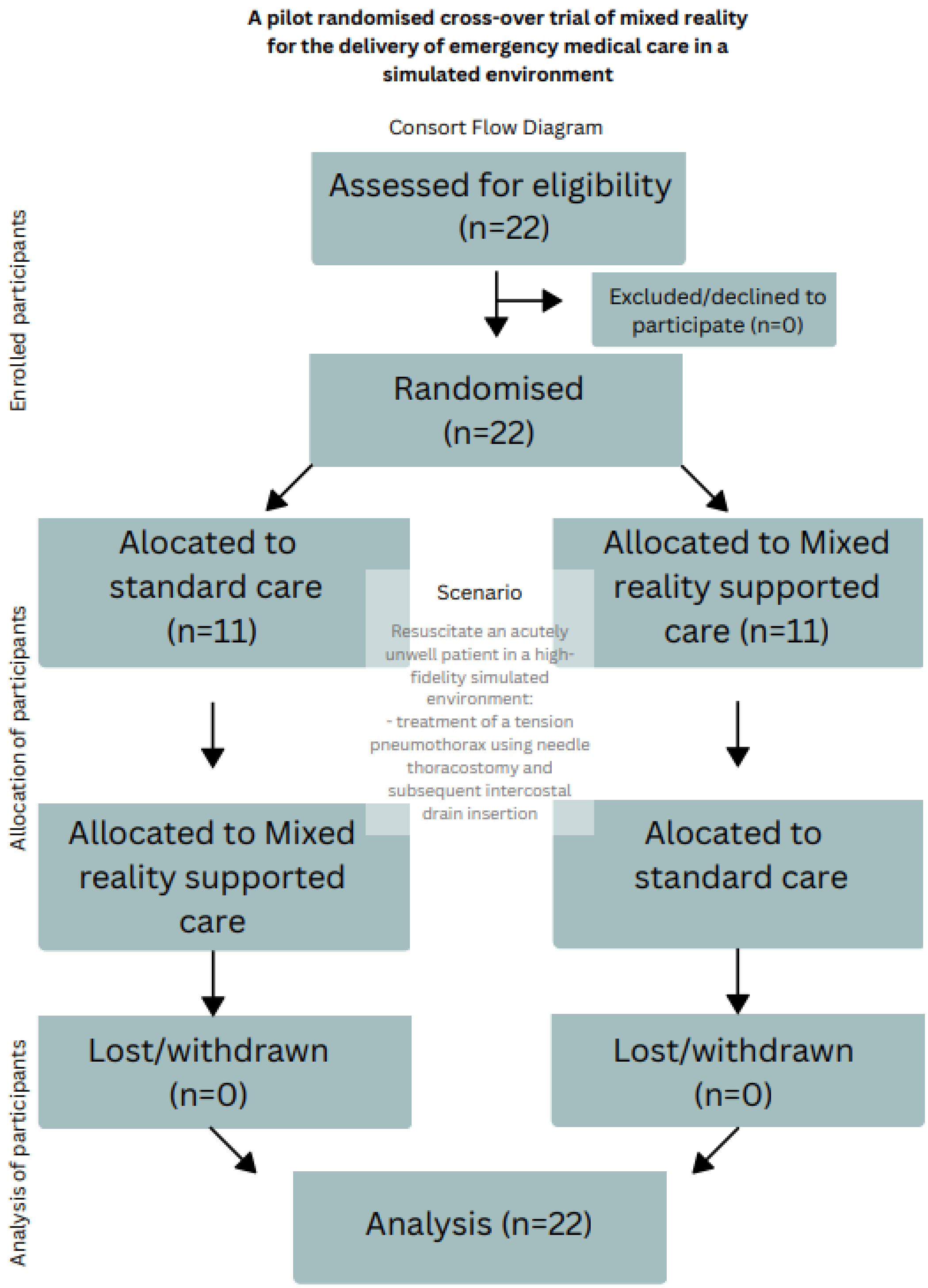

eFigure. Flowchart

© 2023 Lawson J et al. *JAMA Network Open.*

eTable. Clinical Scenarios

**Participant**		Simulator Control
Standard Care Scenario		**Mannequin setup:**
**Candidate Briefing:**<br><br>The country is in the middle of the second surge of the Covid Pandemic. You are doing an ED night shift in a small hospital outside of the city. The only other on-site ED physicians is your registrar who is looking after a patient compromised with severe sepsis and haemodynamically unstable.<br><br>A young lady has walked into ED, she is at university and on returning home after a spin class had chest pain and difficulty in breathing due to the pain. She has never had these symptoms before.<br><br>She has been triaged by the senior resus nurse, who is very concerned as Holly is tachycardic and due to pain is sitting up straight but taking short rapid breaths.<br><br>The ED nurse tells you that the patient has no past medical history and never had any hospital admissions. She asked about drugs and		**Initial Parameters**<br><br>A – Speaking in short sentences as in pain.<br>B – Rapid shallow breathing. RR 40. Uneven breathing pattern. Sats 86% on air<br>C – HR 110bpm SR, BP 90/43<br>D – GCS 15/15. Pain 10/10<br>E – Temp 36.5 Pain is worse when taking deep breaths. Sharp and stabbing in nature, Holly is unable to localise it.

© 2023 Lawson J et al. *JAMA Network Open.*

the patient denies taking anything either medical or recreational. She is unable to elicit any family history as Holly is an adopted child, having lost her parents in a car accident at the age of 2 years. She does not know of any other family.	
**<u>Scenario Progression:</u>**<br><br>Physician to do an A to E assessment but should request full monitoring and oxygen. On examination Holly is breathing rapidly and shallow breaths. She is scared and anxious. On auscultation breath sounds seem quieter on the left but transmitted sounds so difficult to be sure. The physician may request to give the patient some pain relief, provide this.<br><br>Expectation that the physician requests 100% O2 via a non-rebreather mask. Large bore iv access and calls for help.<br>Expectation that an arterial blood gas is performed.<br><br>As examination continues, patient will complain that breathing seems much more difficult and really start to become agitated with hypoxia.<br><br>Monitoring will show a further drop in saturations to 70%. If blood pressure cuff cycling, then SBP 55/35. HR slows to 53bpm SR. Patient developing life-threatening tension pneumothorax.	**<u>Deteriorating Parameters</u>**<br><br>A – Speaking with difficulty<br>B – RR 45 very shallow breathing. Sats 85% even on 100% oxygen<br>C – HR 118bpm SR with VEs. BP 75/45.<br>D – GCS 15/15 E4 V5 M6 agitated due to pain and hypoxia<br><br>ABG on oxygen<br>pH 7.34 pCO2 3.5 pO2 8.5 HCO3 27 BE – 7 Lactate 2.4<br>Hb 123 Na 136 K 4.2 Cl 101 Ca+ 1.2<br><br>Over next two minutes HR 40bpm BP 55/35. Saturating 70%.

© 2023 Lawson J et al. *JAMA Network Open.*

Note any chest manoeuvres are considered an AGP and require full PPE for safety. <br>Proceed to decompress the tension pneumothorax with needle thoracocentesis. This will only have a temporary effect and therefore needs full chest drain insertion. <br>Discussion to proceed to emergency chest drain insertion. Prepare kit necessary and perform procedure. End point is handover.	
**Key confounders** Help does not come immediately on request, as small ED and ED registrar dealing with unstable patient with severe sepsis. *However, will talk on the phone to give advice.*	**Imaging available** Nil
**Key Expected Actions**	**Information**
**Technical Skills**	
• A to E examination • Auscultation of chest • Emergency life-threatening management of tension pneumothorax with needle thoracocentesis.	
**Non-technical Skills**	
• Teamwork with nursing staff, anaesthetic, ICU • Handover/Communication of situation • Situational Awareness	

© 2023 Lawson J et al. *JAMA Network Open.*

• Decision Making	
Debriefing Topics	
Technical Skills	
• Can be discussed after the two arms of the trial having been completed.<br>• Management of tension pneumothorax.	
Non-Technical Skills	
• Ensure psychological safety of the participant.<br>• Escalation<br>• Situational awareness	
Faculty roles	
Briefing: patient	Key tasks:
Mannequin voice<br>You are distressed with your pain. Your pain seems to be all over your chest and is stabbing in nature, it is particularly worse on deep breathing.<br>Never been to hospital before and you do not have any medical history. You are unsure	More and more breathless and to get agitated as breathing is difficult.<br>At the point where the tension pneumothorax is becoming life threatening to become quiet as unable to breath and now significantly hypotensive.

© 2023 Lawson J et al. *JAMA Network Open.*

of your family history as you are an adopted child.<br>Really anxious and you keep calling for your parents to be called.<br>No medical history and no allergies.<br>Not taking any medication.	
Briefing: Nurse	Key tasks:
You are the ED nurse Band 5 doing a locum shift. You are able to follow instruction but not take initiative or prompt the participant.<br><br>You will at some point say to the candidate, that Holly seems unusually tall like your cousins on your mother's side. If this prompt's further questions from the candidate, then you can say my family has a funny syndrome that means they regularly get check-ups, and that you too were tested for Marfan's.<br><br>This prompt is delivered following ABG result return.	Put on monitoring as requested.<br>Attach oxygen when requested to do so.<br><br>Attach iv fluids as requested, you can establish large bore iv access also when requested to do so.<br><br>Assist with equipment for the needle thoracocentesis and chest drain as requested.

© 2023 Lawson J et al. *JAMA Network Open.*

Participant	Simulator Control
Mixed reality - HoloLens2TM Supported care	**<u>Mannequin setup:</u>**
**<u>Candidate Briefing:</u>**<br><br>The country is in the middle of the second surge of the Covid Pandemic. You are doing an ED night shift in a small hospital outside of the city. The only other on-site ED physician is your registrar who is looking after a patient compromised having a STEMI.<br><br>A young man has walked into ED, after sustaining a fall down the stairs at his friend's house. He has been drinking all night and he returned to his friend's house where he planned to spend the night. He got up in the middle of the night to go to the toilet and missed his footing and slipped down the stairs, landing on his right side.<br><br>He has come in because the right side of his chest hurts a lot, and he cannot catch his breath. He has been triaged by the senior nurse and placed in majors. She tells you that she thinks he may have three or four consecutive rib fractures and that he is in pain. She asks you to review him for analgesia.	**<u>Initial Parameters</u>**<br><br>A – Speaking in short sentences as in pain.<br>B – Rapid shallow breathing. RR 40. Uneven breathing pattern. Sats 92% on oxygen via Hudson mask.<br>C – HR 95bpm SR, BP 92/43<br>D – GCS 15/15. Pain 10/10<br>E – Temp 36.5 Pain over ribs 3 – 7 anteriorly and laterally on the right side, with some ecchymosis.

© 2023 Lawson J et al. *JAMA Network Open.*

He has no past medical history and no allergies. He is a cross country runner and is very concerned as he has a big race in 10 days.	
**<u>Scenario Progression:</u>**<br><br>Physician to do an A to E assessment but should request full monitoring. On examination will note a flail segment on the right side with paradoxical breathing pattern. On auscultation will note breathing significant quieter on the right in the apex.<br><br>Expectation that the physician requests 100% O2 via a non-rebreather mask. Large bore iv access and calls for help.<br><br>As examination continues, patient will complain that breathing seems much more difficult and really start to become agitated with hypoxia.<br><br>Monitoring will show a further drop in saturations to 70%. If blood pressure cuff cycling, then SBP 55/35. HR slows to 53bpm SR. Patient developing life-threatening tension pneumothorax.<br><br>***Note any chest manoeuvres are considered an AGP and require full PPE for safety.***	**<u>Deteriorating Parameters</u>**<br><br>A – Speaking with difficulty<br><br>B – RR 45 very shallow breathing. Sats 87% even on 100% oxygen<br><br>C – HR 110bpm SR with VEs. BP 75/45.<br><br>D – GCS 14/15 E4 V3 M6 agitated due to pain and hypoxia<br><br>Over next two minutes HR 53bpm BP 55/35. Saturating 70%.

© 2023 Lawson J et al. *JAMA Network Open.*

Proceed to decompress the tension pneumothorax with needle thoracocentesis. If this is done patient will improve. Start intravenous fluids.<br>Discussion to proceed to emergency chest drain insertion. Prepare kit necessary. End point is handover.	
**Key confounders**<br>Help does not come immediately on request, as small ED and ED registrar dealing with unstable patient having a STEMI. *However, will talk on the phone to give advice.*	**Imaging available**<br>Nil
Key Expected Actions	**Information**
**Technical Skills**	
• A to E examination<br>• Auscultation of chest<br>• Emergency life-threatening management of tension pneumothorax with needle thoracocentesis.	
**Non-technical Skills**	
• Teamwork with nursing staff, anaesthetic, ICU<br>• Handover/Communication of situation<br>• Situational Awareness<br>• Decision Making	
Debriefing Topics	
Technical Skills	

© 2023 Lawson J et al. *JAMA Network Open.*

• Can be discussed after the two arms of the trial having been completed.<br>• Management of tension pneumothorax.	
Non-Technical Skills	
• Ensure psychological safety of the participant.<br>• Escalation<br>• Situational awareness	
Faculty roles	
Briefing: patient	Key tasks:
Mannequin voice<br>You are distressed with your pain, pain on right side of chest, you have mildly slurred speech as you have drunk a lot of alcohol over the last 6 hours with friends.<br>Never been to hospital before.<br>Really worried you need to get fixed up quickly as you are in competition for cross country run in ten days.<br>No medical history and no allergies.<br>Not taking any medication.	More and more breathless and to get agitated as breathing is difficult.<br>At the point where the tension pneumothorax is becoming life threatening to become quiet as unable to breath and now significantly hypotensive.
Briefing: Nurse	Key tasks:

© 2023 Lawson J et al. *JAMA Network Open.*

You are the ED nurse Band 5 doing a locum shift. You are able to follow instruction but not take initiative or prompt the participant.	Put on monitoring as requested.<br>Attach oxygen when requested to do so.<br><br>Attach iv fluids as requested, you can establish large bore iv access also when requested to do so.<br><br>Assist with equipment for the needle thoracocentesis.

© 2023 Lawson J et al. *JAMA Network Open.*

eAppendix. Questionnaire

Imperial College London

HoloLens Participant Questionnaire *(will be delivered electronically through Qualtrics)*

HoloLens User Survey

Start of Block: Introduction

We would be very grateful if you could complete this quick survey so we can better understand your experience of using the Microsoft HoloLens and how it has impacted your daily work. All responses are anonymous.

End of Block: Introduction

Start of Block: About you

Q1 How would you describe your gender? ◯ Male

(including transgender men) (1) ◯ Female

(including transgender women) (2) ◯ Prefer to

self-describe as ________________ (3) ◯ Prefer

not to say (4)

Q2 What is your age?

◯ 18 - 24 (1)

◯ 25 - 34 (2)

◯ 35 - 44 (3)

◯ 45 - 54 (4)

◯ 55 - 64 (5)

◯ 65+ (6)

© 2023 Lawson J et al. *JAMA Network Open.*

Imperial College London

Q3 What is your job role? ○ Doctor

- Consultant/SpR (1) ○ Doctor

- SHO/FY1 (2) ○ Nurse (3)

○ Allied Health Professional (4) ○ Medical Student (5) ○

Healthcare Assistant (6) ○ Other (7)

____

Q4 What is your previous experience with this type of device (e.g. Microsoft HoloLens, Google Glass, Samusung Gear VR)?

- ☐ I own a device (1)
- ☐ I have previously used such a device in a healthcare setting (2)
- ☐ I have previously used such device in a non-healthcare setting (3)
- ☐ I have heard of these devices, but never seen or used one (4)
- ☐ I have never heard of this technology (5)

Q5 What is your experience of using video conferencing/telemedicine software at work (e.g. Microsoft Teams, Zoom, Google Meet)?

- ☐ I used them prior to COVID-19 (1)
- ☐ I have only used them since their introduction during COVID-19 (2)
- ☐ I have never used them (3)

© 2023 Lawson J et al. *JAMA Network Open.*

Imperial College London

- [ ] I use them occasionally (4)
- [ ] I use them regularly (5)

End of Block: About you

Start of Block: Your experience of using the HoloLens

Q6 How did you use HoloLens during this scenario?

- [ ] I was wearing the HoloLens for conducting clinical care (1)
- [ ] I was a member of the remote team with someone else wearing the device (2)

Q7 Which functions of the technology did you use?

- [ ] Video / voice communication (1)
- [ ] Viewing electronic health record data (e.g. blood results, observations) (2)
- [ ] Viewing imaging (3)
- [ ] Using the interactive tools (e.g. drawing on the screen) (4)
- [ ] Other (5) ____

Q8 If you have worn and used the HoloLens please rank these statements

© 2023 Lawson J et al. *JAMA Network Open.*

Imperial College London

	Strongly agree (1)	Agree (2)	Somewhat agree (3)	Neither agree nor disagree (4)	Somewhat disagree (5)	Disagree (6)	Strongly disagree (7)
The HoloLens is easy to set up and use (1)	○	○	○	○	○	○	○
The HoloLens is easy to wear with PPE (2)	○	○	○	○	○	○	○
The HoloLens is comfortable to wear (3)	○	○	○	○	○	○	○
The audio quality is good (4)	○	○	○	○	○	○	○
The video quality is good (5)	○	○	○	○	○	○	○
The quality of virtual objects (e.g. X-Rays, Cerner data) is good (6)	○	○	○	○	○	○	○
I did not have any problems with the device when using it (7)	○	○	○	○	○	○	○
Before using the device I thought it would not be useful (8)	○	○	○	○	○	○	○
The network connection was good all the time (9)	○	○	○	○	○	○	○

© 2023 Lawson J et al. *JAMA Network Open.*

I can see the benefit in using this technology for delivering clinical care (10)	○	○	○	○	○	○	○

Q9 If you have used the technology to communicate with someone wearing a HoloLens please rank these statements

	Strongly agree (1)	Agree (2)	Somewhat agree (3)	Neither agree nor disagree (4)	Somewhat disagree (5)	Disagree (6)	Strongly disagree (7)
I find the technology easy to set up and use (1)	○	○	○	○	○	○	○
The video quality is good (2)	○	○	○	○	○	○	○
The audio quality is good (3)	○	○	○	○	○	○	○
I am able to easily place and manipulate virtual objects (e.g. XRays, Cerner Data) (4)	○	○	○	○	○	○	○
I did not have any problems when using the technology (5)	○	○	○	○	○	○	○

© 2023 Lawson J et al. *JAMA Network Open.*

Imperial College London

The network connection was good all the time (6)	○	○	○	○	○	○	○
I can see the benefit in using this technology for delivering clinical care (7)	○	○	○	○	○	○	○

End of Block: Your experience of using the HoloLens

Start of Block: The impact of using HoloLens on the quality of care and communication

Q10 Please rank these statements with regards to the impact of HoloLens on delivering clinical care

	Strongly agree (1)	Agree (2)	Somewhat agree (3)	Neither agree nor disagree (4)	Somewhat disagree (5)	Disagree (6)	Strongly disagree (7)
I could provide care more quickly when using the HoloLens (1)	○	○	○	○	○	○	○
The HoloLens adds value (2)	○	○	○	○	○	○	○
I feel my team would be safer when using the HoloLens for looking after COVID patients (3)	○	○	○	○	○	○	○
HoloLens improves the quality of patient care (4)	○	○	○	○	○	○	○

© 2023 Lawson J et al. *JAMA Network Open.*

The HoloLens should be used all the time (5)	○	○	○	○	○	○	○
Care is delivered more efficiently when using HoloLens (6)	○	○	○	○	○	○	○
I would be happy to use the HoloLens again (7)	○	○	○	○	○	○	○
The HoloLens improved the experience of delivering care (8)	○	○	○	○	○	○	○
I am able to make better clinical decisions when using the HoloLens (9)	○	○	○	○	○	○	○

Q11 Please rank these statements with regards the impact of HoloLens on communication and teamwork

	Strongly agree (1)	Agree (2)	Somewhat agree (3)	Neither agree nor disagree (4)	Somewhat disagree (5)	Disagree (6)	Strongly disagree (7)
HoloLens improves the quality of communication (1)	○	○	○	○	○	○	○
HoloLens improves teamwork (2)	○	○	○	○	○	○	○
HoloLens provides me with better situational awareness (3)	○	○	○	○	○	○	○

© 2023 Lawson J et al. *JAMA Network Open.*

I feel I am more engaged in care delivery and decisions when using the HoloLens (4)	○	○	○	○	○	○	○
I am better able to relay information to my team when using HoloLens (5)	○	○	○	○	○	○	○
HoloLens provides me with a "better feel" of what is going on with the patient (6)	○	○	○	○	○	○	○

End of Block: The impact of using HoloLens on the quality of care and communication

---

Start of Block: Final comments

Q12 What is the most positive aspect of the HoloLens?

____

Q13 What is the most negative aspect / thing you would change about the HoloLens?

____

Q14 Please report any safety concerns or problems you had with the headset

____

Q15 Please add any other comments you have regarding the HoloLens

____

End of Block: Final comments

© 2023 Lawson J et al. *JAMA Network Open.*
