# Supplementary material for: Effect of Mixed Reality on Delivery of Emergency Medical Care in a Simulated Environment: A Pilot Randomized Crossover Trial
Source: JAMA Netw Open. 2023 Aug 28;6(8):e2330338. doi: 10.1001/jamanetworkopen.2023.30338 (PMC10463095; doi:10.1001/jamanetworkopen.2023.30338)
Supplement: Supplement 2. — Trial Protocol and Statistical Analysis Plan [file jamanetwopen-e2330338-s002.pdf]

Ministry of Defence Research Ethics Committee (MODREC)

PROTOCOL

HoloLens for the delivery of distributed medical care during COVID-19 and beyond - a pilot randomised cross-over trial of HoloLens2 for the delivery of distributed medical care during COVID-19 in a simulated environment

MODREC Reference: 1083/MODREC/20
IRAS ID - 298084

This protocol describes the proposed study and provides information about procedures for entering participants. Every care was taken in its drafting, but corrections or amendments may be necessary. These will be circulated to investigators in the study. Problems relating to this study should be referred, in the first instance, to the Chief Investigator.

This study will adhere to the principles outlined in the UK Policy Framework for Health and Social Care Research. It will be conducted in compliance with the protocol, the Data Protection Act, and other regulatory requirements as appropriate.

Application No: 1083/MODREC/20

Ministry of Defence

Ministry of Defence Research Ethics Committee (MODREC)

MODREC Application Form

Please ensure the Research Sponsor checklist has been completed and this application has received a SAC approval prior to applying to MODREC.

The Research Sponsor checklist and any other supporting documents must be included as annexes to the main body of the application. Once the application has been completed in full, the guidance text in blue italics can be deleted and the application emailed to the MODREC Secretariat (MODREC@dstl.gov.uk)

1. Study Title (including any abbreviated titles)

A pilot randomised cross-over trial of HoloLens2™ for the delivery of distributed medical care during COVID-19 in a simulated environment.

2. Date/Version

**Date:** 14/12/2020	**Version:** 4

3. Summary of Project

This pilot randomised cross-over study aims to assess the technical feasibility of a novel Mixed Reality (MR) headset known as the HoloLens 2™ (HL2) for the delivery of direct clinical care in simulated COVID-19 contaminated environments. The HL2 is an untethered holographic computer that is worn as a headset. Using software called 'Remote Assist' it permits a 'hands free' scalable telemedicine platform which can be used without the requirement for touch or interaction with a physical computer.

We will prospectively evaluate the impact of the device in the management of a simulated COVID-19 clinical resuscitation scenario. The study will be conducted according to a randomised cross-over design with participants completing two simulated clinical scenarios. Participants will be randomly assigned to one of two groups - standard care, or HL2 supported care – for the first scenario prior to cross-over. The primary outcome measure will be error rate as assessed by the validated ICECAP multi-dimensional error capture tool. Secondary outcomes pertaining to scenario completion, teamwork, PPE use, stress/cognitive load and user acceptability of the HL2 device will also be recorded. The study will be undertaken in a specialist clinical simulation facility facilitated by experienced simulation practitioners.

4. Investigators

4a. Chief Investigator and Study Coordinator

Name and Title: Mr James Kinross (PhD FRCS)

Grade/Rank: N/A

Post Title: Senior Lecturer in Surgery and Consultant Surgeon

Department: Department of Surgery and Cancer

Establishment:

Address:

Telephone:

Email:

https://www.imperial.nhs.uk/consultant-directory/james-kinross

4b. Does this project contribute towards a qualification? No

4c. Other Investigators/Collaborators/External Consultants

Mr Guy F J Martin, Imperial College, Clinical lecturer General Surgery (https://www.imperial.ac.uk/collegedirectory/index.asp?PeopleID=528190)

Dr Phillip Pratt, Imperial College, Faculty of Medicine, Department of Surgery & Cancer (https://www.imperial.ac.uk/people/p.pratt)

4d. Name of the Volunteer Advocate or Independent Medical Officer

Jo Burns, Head of Clinical Research Operations (maternity cover)

Address:

Telephone:

Email:

https://www.imperial.ac.uk/research-and-innovation/support-for-staff/joint-research-office/icht-studies/

5. Research Sponsor

Imperial College London is the main research Sponsor for this study. For further information regarding the sponsorship conditions, please contact the Head of Regulatory Compliance.

Research Governance and Integrity Team
Imperial College London and Imperial College Healthcare NHS Trust
Room 215, Level 2, Medical School Building
Norfolk Place
London, W2 1PG
Tel: 0207 594 9459/ 0207 594 1862
http://www3.imperial.ac.uk/clinicalresearchgovernanceoffice

6. Preferred Timetable

6a. Preferred Start Date: 1st May 2021

6b. Expected Date of Completion: End of study definition is final participant visit

7. Other Organisation(s) Involved and Funding

7a. Department/Organisation Requesting Research (if applicable):
Imperial College London

7b. If you are receiving funding, please provide details here: MOD Chief Scientific Advisor via Defence Science and Technology Laboratory (Dstl) and the Defence and Security Accelerator (DASA).

7c. Please declare any competing or conflicts of interests: None

7d. Type of research

i. Equipment

8. **Scientific Assessment Approval**

8a. Name of SAC that has reviewed/approved this application: Dstl SAC

8b. Date of SAC approval:

8c. SAC reference number: 1083/MODREC/20

9. Purpose of the Study and Defence Benefit

Telemedicine encompasses a wide range of technologies that support the delivery of remote care, and the use of such technologies has been identified as a key potential innovation in the response to COVID-19;(1) providing a means to efficiently deliver care and leverage access to multiple remote specialists whilst simultaneously protecting staff and patients from exposure to the virus.(2, 3) There is an opportunity for technology to not only bring additional benefits and scope to clinical care, but also to simultaneously act as personal protective equipment which may also act to reduce the risk of staff infection. During COVID-19 shortage of PPE has been a national emergency.(3) Protecting the health and safety of healthcare workers is vital to maintain the quality of care delivered to individual patients and the ability of health systems to deliver care at scale. In Italy up to 20% of healthcare workers became infected with the virus during the initial phase of the pandemic,(4) and a number of them died; a picture that is seen across the globe and in the UK. As such, there is a pressing need to reduce the risk of transmission of all pathogens transmitted from patients to healthcare workers via all practical means.

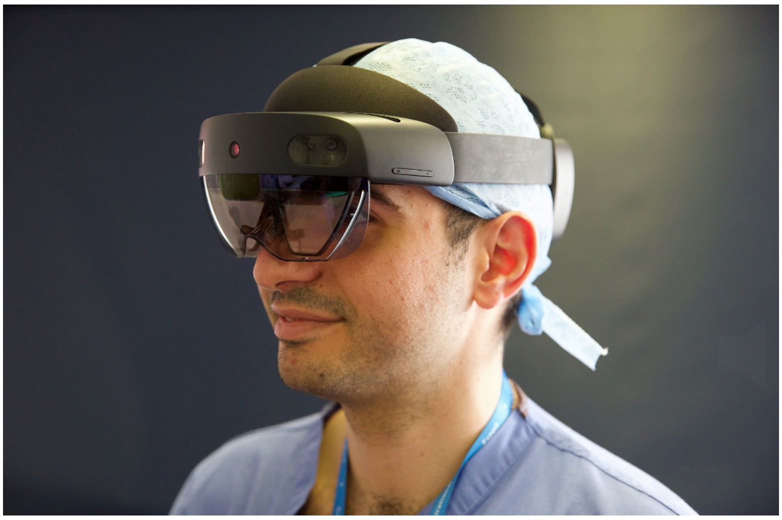

Figure 1. The HoloLens 2 Headset. It consists of a mounted computer (at rear of the head), a visor which projects holographic images on the user's eye. It also contains a forward-looking camera in the centre of the headset and sensors mounted at the front on each side. Finally, it possesses a noise cancelling microphone for voice communication.

HoloLens 2™ is an MR head-mounted device with a range of functionality (figure 1). The device is developed and marketed by Microsoft Corporation (Redmond, WA, USA). The headset combines several types of sensors (infra-red time-of-flight cameras, high definition RGB cameras, accelerometers and microphones), and provides a true heads-up display functionality with the ability to place 2D and 3D objects within a user's visual field, whilst also delivering live bidirectional communication via video. Mixed reality composites and voice can also be shared with multiple remote users via the Remote Assist application (figure 2a and b). The HoloLens™ has been used in a variety of clinical scenarios including perioperative planning, surgical training and 3D telemedicine support.(5-7)

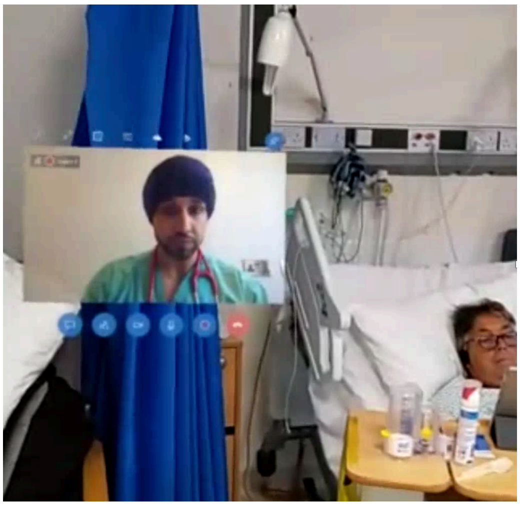

Figure 2a
HL2 wearer can visualise colleagues
Situated remotely facilitating a bedside MDT

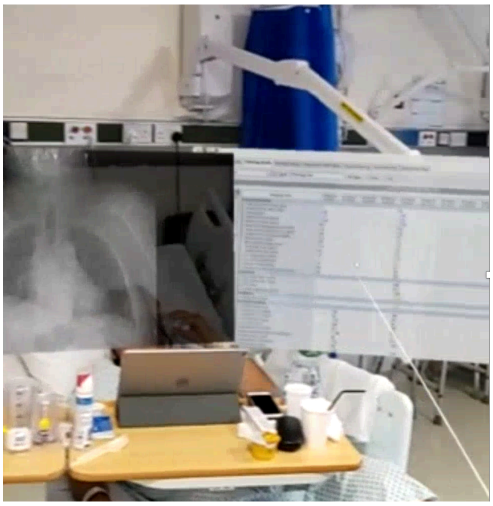

Figure 2b
The wearer can also visualise imaging and electronic healthcare records 'heads up'.

The HL2 system therefore has significant potential for defence benefit. Specifically:

1) Reduction of COVID-19 transmission risk and strengthening of staff safety by reducing need for team exposure to high-risk environments (figure 2a).
2) Improved heads-up situational awareness and data visualisation - electronic healthcare record and imaging data can be projected into the field of view, or objects virtually marked and illustrated in the real world by remote members of the clinical team (figure 2b). Moreover, data can be captured and streamed back to wider team by the end-user in the field, who also has the ability to virtually mark targets or objects of interest.

3) Support the provision of remote specialist access. Specifically, Remote Assist will aid the delivery of care by permitting multidisciplinary teams to function across wide geographical areas.
4) Deliver remote immersive education and training - all first-person views and MR content can be captured and securely streamed for educational and training use either in real-time or on-demand.
5) Generation of new knowledge and capabilities - the storage of all MR content captured through the device has the potential to create a rich repository of data from which future insights and technology developments can be leveraged through human or machine learning based analysis.

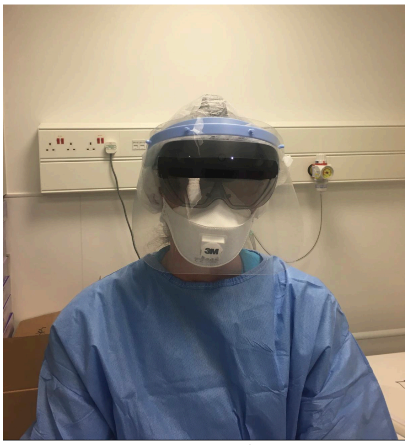

Figure 3: Adaption of standard PPE for use with the HL2 system. A strip of plastic has been removed at the front of the visor.

Despite this potential, the system has yet to be formally assessed in a structured manner and therefore the benefit of MR over standard clinical practice in clinical scenarios has yet to be fully assessed, and the true value proposition has yet to be defined. It is also still unclear how the lack of physical presence of a provider impacts the teamwork, communication, and workload of a clinical team e.g. during resuscitation.

The HoloLens2™ has been deployed to support the delivery of clinical care during COVID-19, and the early impact of its use has been reported by this research team as part of a quality improvement study at Imperial College London.(8) This demonstrated that it reduced the frequency of potential COVID-19 contacts by 80% during daily clinical ward rounds because it reduced the number of team members that needed to enter a COVID-19 environment at any one time. This in-turn reduced PPE consumption rates. The benefit of the system was further improved because it can be controlled through the use of voice command or hand gestures which significantly reduces the need for physical contact with computer equipment and therefore the transmission of nosocomial infections. However, robust trial evidence is lacking and the exact mechanisms through which the system influences team performance are not defined. This is required before this technology can be deployed at scale within a military operational environment.

The HL2 system is a commercial device not designed for use in challenging or high- risk clinical environments. Therefore, in future it will be necessary to adapt standard PPE visors for use with the system. This is because the technology's sensors and cameras must be unimpeded by plastic (figure 3). Therefore, novel approaches are required that seamlessly allow the headset to be adopted when PPE is worn. These can be developed using a combination of 3D printed attachments for standard plastic visors. Whilst the commercial device is a relatively mature technology, further development work supported by this study such as the design of bespoke accessories to support compliance with infection prevention and control when working with aerosol generating procedures, and also further software development to support a more functional and user-centric clinical interface is required.

Work undertaken to support the delivery of this study:

The study group is undertaking a technology development project to support the delivery of this study. The aim of this development stage is to establish the technical feasibility of using HoloLens™ with a 3D printed bespoke visor, and to develop and test standard processes for the secure collection and analysis of mixed reality outputs to support improvements in

technology deployment and clinical care. This work is being undertaken in two distinct work packages.

1 - Development of a bespoke 3D-printed face visor and linked accessories:

We are creating a bespoke 3D printed face visor and linked accessories that permits the sensors and cameras of the HoloLens™ to continue to function whilst simultaneously providing adequate protection, particularly during AGPs in COVID-19 environments. An initial design phase with observations of clinical use of HoloLens™ headsets and interviews with frontline clinicians who may have to use the device in a range of clinical environments has been undertaken to define the ideal requirements for a future HoloLens™ protective visor and other relevant linked accessories such as light sources or additional audio equipment. This will inform the design and production of device specific protective equipment and accessories.

2 - Software development and inter-operability

The MR content generated through the HoloLens™ has the potential to provide an enormously rich library of data that can inform better clinical practice, provide more effective medical education and support the development of new technologies. A key aim of this aspect of the supporting work is to provide a means to capture, store and annotate MR data captured during routine clinical interactions in a user-friendly method in order to curate a digital data repository that can support future clinical care, education and research.

References:

1. Hollander JE, Carr BG. Virtually Perfect? Telemedicine for Covid-19. N Engl J Med. 2020.
2. Turer RW, Jones I, Rosenbloom ST, Slovis C, Ward MJ. Electronic Personal Protective Equipment: A Strategy to Protect Emergency Department Providers in the Age of COVID-19. J Am Med Inform Assoc. 2020.
3. The L. COVID-19: protecting health-care workers. Lancet. 2020;395(10228):922.
4. Remuzzi A, Remuzzi G. COVID-19 and Italy: what next? Lancet. 2020;395(10231):1225-8.
5. Zuo Y, Jiang T, Dou J, Yu D, Ndaro ZN, Du Y, et al. A Novel Evaluation Model for a Mixed-Reality Surgical Navigation System: Where Microsoft HoloLens Meets the Operating Room. Surg Innov. 2020;27(2):193-202.
6. Pratt P, Ives M, Lawton G, Simmons J, Radev N, Spyropoulou L, et al. Through the HoloLens looking glass: augmented reality for extremity reconstruction surgery using 3D vascular models with perforating vessels. Eur Radiol Exp. 2018;2(1):2.
7. Tepper OM, Rudy HL, Lefkowitz A, Weimer KA, Marks SM, Stern CS, et al. Mixed Reality with HoloLens: Where Virtual Reality Meets Augmented Reality in the Operating Room. Plast Reconstr Surg. 2017;140(5):1066-70.
8. Martin G, Koizia L, Kooner A, Cafferkey J, Ross C, Purkayastha S, et al. Use of the HoloLens2 Mixed Reality Headset for Protecting Health Care Workers During the COVID-19 Pandemic: Prospective, Observational Evaluation. J Med Internet Res. 2020;22(8):e21486.

10. Study Design, Method and Data Analysis

Aims and objectives

To prospectively assess the impact of HoloLens 2™ in a simulated COVID-19 setting for the assessment and resuscitation of critically unwell patients.

There are no secondary objectives for this study.

Hypothesis

Remote care delivered with the HL2 is equivalent to standard clinical practice for team performance, workload and technical performance.

PROJECT DESIGN

Because the evolution of the COVID-19 pandemic is unpredictable we will utilise a simulated environment to establishing our core hypothesis about the effectiveness of MR telemedicine.

This is a prospective observational simulated trial designed to evaluate the impact of MR telemedicine technology (HL2) on care delivered during a simulated adult resuscitation.

Recruitment: Study participants will be recruited from a convenience sample of clinical staff who provide care to COVID-19 patients at Imperial College Healthcare NHS Trust and Imperial College London. This will include doctors in training and equivalent non-training medical staff, in addition to independent specialist nurse practitioners. All participants will have prior experience/qualifications in advanced life support skills and resuscitation. Informed consent will be obtained prior to commencing the study.

Quality assurance and standardisation: All team members will receive standardised training in two components of practice prior to entry into the study in order to familiarise themselves with the HL2 device and ensure basic technical competency:

1. HL2 operation: All participants will receive a standardised tutorial on how to operate the HL2 headset. This 15-minute practical induction will provide them fundamentals of use, namely a) how to wear PPE with the device b) how to turn on the device, access software and interact with the device c) how to make and receive calls and use clinical data. The training will be run as a hands-on demonstration/run-through clinical scenario provided by the study team in which participants will be able to familiarise themselves with the HL2 device and its use. All participants will be expected to perform a simple competency test before being allowed to proceed to the trial phase to ensure standardised basic technical competency and mitigate device related ‘first-use’ learning effects.
2. Advanced Life Support: All participants will be provided with a refresher instruction on core aspects of advanced life support according Resuscitation Council UK guidelines. Instruction provided will cover all knowledge and practical skills required to successfully complete each of the simulated clinical scenarios, including advanced airway skills. Clinical scenario protocols will be written by experienced simulation practitioners and based upon the guidelines for the management of in-hospital assessment and resuscitation of the critically unwell patient.

Simulation study method: The study will be conducted according to a randomised cross-over design. Participants will complete two simulated clinical resuscitation scenarios as part of a single half-day study session that will also include their standardised training. There will

be a wash-out period of at least 1 hour between each scenario. This is a deliberately pragmatic methodological approach that has been chosen to ensure sufficient participant recruitment and study completion given time demands on participants if the study were to be split across multiple sessions. Prior to commencing the first simulated scenario participants will be randomly assigned to one of two groups - standard care, or HL2 supported care - and will cross-over into the alternate group for the second scenario. The order of the test scenarios will be counterbalanced to mitigate any learning effects. The order of HL2/standard care will be determined by using sealed envelopes prior to starting the simulation. Participants will be required to undertake advanced airway skills in order to test the impact of HL2 on remotely supporting the delivery of vital clinical skills in high stress situations. The clinical scenarios will not be identical but will be based upon standardised clinical moulages undertaken for basic and advanced life support qualifications, and those undertaken by doctors in training as part of their core curriculum to ensure consistency, reliability and control for learning effects.

Group 1 (Standard care) - the participant will act as a first responder to a critically unwell patient with suspected COVID-19. They will be required to make an initial assessment, perform advanced life support and handover the patient to a senior clinician played by a member of the study team for successful completion of the scenario. The same study team member will act as the senior clinician for both study scenarios to reduce variability. A nurse, played by a member of the study team, will be provided to assist at the bedside, and senior clinical support will be available by telephone if required. This senior clinical support will again be standardised across all scenarios and provided by a member of the study team. Relevant clinical data and patient information will be available via a standard desktop electronic healthcare record system. Resuscitation algorithms and standard operating procedures will be provided on the desktop computer.

Group 2 (HL2 supported care) - in addition to standard care participants will complete the scenario whilst wearing an HL2 headset. A senior clinician, standardised across scenarios and played by a member of the study team to ensure consistency, will be available to talk and interact directly with the participant using voice and mixed reality prompts via the HL2 throughout the simulated scenario. The participant will be able to request 'heads-up' views of patient information and clinical data, in addition to resuscitation algorithms and standard operating procedures via the HL2 headset. All other standard material and equipment as per Group 1 will also be provided.

The simulation will be undertaken in a specialist simulation lab at Imperial College London facilitated and led by experienced simulation practitioners. The research team will be solely responsible for capturing observational data. A high-fidelity adult patient simulator (Laerdal) will be used to simulate an unwell adult with COVID-19. Vital signs will be standardized and pre-programmed with an adjustable override feature controlled by the study investigators. Before the start of each simulation session, study investigators will provide information to the participants on the patient using a standardised clinical moulage, with instructions to follow during the case, and the roles of study participants defined prior to the start of the simulation. Simulated medications, fluids, and equipment will all be available. Full PPE will also be available to all participants in the scenario. All scenarios will be video recorded, and these will subsequently be assessed by two independent researchers.

We aim to enrol 30 individual participants into the study who will undertake a total of 60 clinical scenarios (30 as standard care, 30 as HL2 supported care). The study will take place in a single setting - the study team anticipate this will last a maximum of 2hrs to allow for consent, initial technical training and the completion of 2x clinical scenarios.

Outcome measures:

The primary outcome will be

- Error rate as assessed by the validated ICECAP Tool (Imperial College Error Capture Tool) a validated multi-dimensional assessment of error

Secondary outcomes will be

- Time to complete the clinical scenario
- Successful completion of the clinical scenario - as assessed by simulation team
- Global assessment of team performance - assessed using the validated OTAS Tool (Observational Teamwork Assessment for Surgery) and T-SAW-C Tool (Teamwork Skills Assessment for Ward Care)
- Errors in PPE use - assessed according to Public Health England (PHE) guidance and number of visor failures (e.g., inadvertent removal, misting, contamination or obstruction of clinical care)
- Participant stress/cognitive load - assessed via the validated NASA Task Load Index (NASA-TLX) and State Trait Anxiety Index (STAI) Tools
- Usability / technology feedback questionnaire for those wearing the HL2

All questionnaire items and assessment tools listed above are provided in the attached documents. Participant responses will be collected at the time of study completion, and assessment of video recordings will be undertaken during the data analysis stage of the study

Statistical analysis: Standard descriptive data will be employed. Independent two-sample t-tests will be calculated for normal continuous data, including ICECAP and OTAS scores, and time-to completion data. The Wilcoxon-Mann-Whitney U test will be used to analyse TLX scores. Multivariate linear regressions will be calculated to control for potential confounders and to assess association. Within subject comparisons will also be made to assess how the technology influences individual performance.

11. Safety

11a. How will the safety of the research be managed?

All participants and study team members will follow both the Imperial College London and Imperial College Healthcare NHS Trust guidance on safe clinical practice during COVID-19. All participants will be asked to complete a screening questionnaire and will wear appropriate PPE when visiting the simulation lab as per local guidelines. The current version of the screening questionnaire in use at the Trust is provided in Appendix 1. These guidelines are subject to change and the current version will be strictly adhered too.

While performing simulations, full barrier PPE will be worn. Participants will be kept separated when completing questionnaires and there will be no congregation of staff. We will keep a register or attendees for tracking and trace purposes. The study will take place in a purpose-built clinical simulation lab at Imperial College London. All local health and safety policies and procedures will be adhered to throughout.

11b. Who is the named person taking responsibility for the overall safety of the research, and who will be responsible for day-to-day safety?

Mr James Kinross – Chief Investigator

11c. How will the researchers conducting this study be made aware of:

i. Their responsibilities for reporting any new safety issues which arise after the start of the project, and

ii. Their responsibilities for reporting adverse events in the conduct of the project?

The scientific committee will provide clear guidance to all researchers on safety reporting mechanisms and their responsibilities. These will be provided in a written information sheet and they will be reinforced during all simulation briefings and debriefings. The greatest risk will be the transmission of COVID-19 between participants. We will ensure that this is incorporated into the briefing and that all members of the team have a clear reporting line in the event of a positive test as per Imperial College London / Imperial College Healthcare NHS Trust policies and procedures. We will minimise the number of participants together at any one time and will ensure that all safe working practice regulations (e.g., appropriate social distancing, following maximum room capacity rules, use of PPE) are strictly adhered to so that the study in conducted in a "COVID secure" manner as per local rules and regulations.

11d. Adverse Events

Adverse Event (AE): is any untoward medical occurrence in a patient or clinical study subject.

Serious Adverse Event (SAE): any untoward and unexpected medical occurrence or effect that:

- Results in death
- Is life-threatening – refers to an event in which the subject was at risk of death at the time of the event; it does not refer to an event which hypothetically might have caused death if it were more severe
- Requires hospitalisation, or prolongation of existing inpatients' hospitalisation
- Results in persistent or significant disability or incapacity
- Is a congenital anomaly or birth defect

Medical judgement should be exercised in deciding whether an AE is serious in other situations. Important AEs that are not immediately life-threatening or do not result in death or hospitalisation but may jeopardise the subject or may require intervention to prevent one of the other outcomes listed in the definition above, should also be considered serious.

Reporting Procedures:
All adverse events should be reported. Depending on the nature of the event the reporting procedures below should be followed. Any questions concerning adverse event reporting should be directed to the Chief Investigator in the first instance.

Non serious AEs
All such events, whether expected or not, should be recorded- it should be specified if only some non-serious AEs will be recorded, any reporting should be consistent with the purpose of the trial end points.

Serious AEs
An SAE form should be completed and emailed to the Chief Investigator within 24 hours. However, relapse and death due to and hospitalisations for elective treatment of a pre-existing condition do not need reporting as SAEs.

All SAEs should be reported where in the opinion of the Chief Investigator, the event was:

- 'related', ie resulted from the administration of any of the research procedures; and
- 'unexpected', ie an event that is not listed in the protocol as an expected occurrence

Reports of related and unexpected SAEs should be submitted within 15 days of the Chief Investigator becoming aware of the event, using the NRES SAE form for non-IMP studies. The Chief Investigator must also notify the Sponsor of all related and unexpected SAEs.

Local investigators should report any SAEs as required by their Local Research Ethics Committee, Sponsor and/or Research & Development Office.

Contact details for reporting SAEs:

RGIT@imperial.ac.uk

Chief Investigator: j.kinross@imperial.ac.uk

12. Ethical Considerations

ETHICS APPROVAL
The Study Coordination Centre has obtained approval from the Ministry of Defence Research Ethics Committee (MODREC) and Health Regulator Authority (HRA). The study must also receive confirmation of capacity and capability from each participating organisation before accepting participants into the study or any research activity is carried out. The study will be conducted in accordance with the recommendations for physicians involved in research on human subjects adopted by the 18th World Medical Assembly, Helsinki 1964 and later revisions.

CONSENT AND CONFIDENTIALITY
Individual informed consent for the capture, storage and analysis of HoloLens™ MR data and scenario recordings will be obtained from all participants as required. It will be the responsibility of local collaborators to obtain written informed consent from each participant after adequate explanation of the project, potential hazards and process for data capture and storage. The original copy of the signed and dated informed consent must be retained at the participating organisation and is subject to inspection by representatives of the Sponsor, or representatives from Regulatory Authorities. The standardised information sheet and consent form is included in this protocol. The Chief Investigator will preserve the confidentiality of participants taking part in the study and is registered under the Data Protection Act.

INDEMNITY
Imperial College London holds negligent harm and non-negligent harm insurance policies which apply to this study.

SPONSOR
Imperial College London will as the main sponsor for this study

DATA STORAGE AND RETENTION
All data will be stored for a minimum of 10 years (or according to changes in regulatory requirements). Data generated by this work will be processed in accordance with the Data Protection Act 2018. Retention and analysis will be conducted with regard to all local policies relating to the collection, holding and disclosure of data relating to individuals. The Principal and Co-applicants will act as custodians of the data and be responsible for its security. The CI will ensure the continued storage of all relevant data and documentation, even if they leave the clinic/practice or retire before the end of the required storage period. Delegation will be documented in writing.

AUDITS

The study may be subject to inspection and audit by Imperial College London under their remit as sponsor and other regulatory bodies to ensure adherence to GCP and the UK Policy Framework for Health and Social Care Research.

13. Participants

13a. Number of Participants: 30

13b. Lower Age Limit: 18

13c. Upper Age Limit: No restrictions

13d. Birth Sex (male/female): No restrictions.

13e. Please provide justification for the sample size, and age/sex restrictions: It is not possible to perform a power calculation for this work, as there are currently no data available on HL2 interventions upon which a standard deviation of the primary outcome measure can be calculated. The objective of this pilot study is therefore to determine a power calculation for a formal randomised control trial in patients.

14. Selection Criteria

14a. List your participant inclusion criteria:
Clinical staff who provide care to COVID-19 patients at Imperial College Healthcare NHS Trust and Imperial College London. This will include doctors in training and equivalent non-training grade medical staff, in addition to independent specialist nurse practitioners. All participants will have prior experience/qualifications in advanced life support skills and resuscitation. Subjects should be 18 or above.

14b. List your participant exclusion criteria:
Those already independently competent in advanced airway management
Unable to consent
Unable to wear MR headset – either because of head shape or because of problems with nausea or previous challenges with VR.
Unwilling or unable to take part

15. Recruitment

15a. Describe how potential participants will be identified:

The study is being undertaken at a single institution. Potential participants will be individuals who have already undertaken a simulation programme in life support skills at the Trust or College. They will be identified through the Medical Education Department at Imperial College Healthcare NHS Trust who already have an established program of simulation training, including a specific focus on COVID-19 preparation and resilience building. This process will be undertaken in conjunction with the Director of Medical Education. The organisation has an established track record of providing simulation training in addition to the evaluation of these educational programs; standard local practices for conducting such work will be followed throughout the conduct of this study.

15b. Describe how potential participants will be approached:
They will be approached via the Medical Education Department or Simulation Team from Imperial College Healthcare NHS Trust / Imperial College. Participants will also be approached at hospital educational events. Example wording for the content of

recruitment material is provided in Appendix 2; all final materials will be subject to local Trust and College approval at the time of recruitment.

15c. Describe how potential participants will be recruited:

Participants will email the CI if they wish to be involved. They will then be sent information sheets and the consent form. If they are still interested, they will then be able to contact and speak to the CI and be given a verbal briefing.

16. Consent

16a. Describe the process you will use when seeking and obtaining consent:
Consent to enter the study must be sought from each participant only after a full explanation has been given, an information leaflet offered, and time allowed for consideration. All participants will receive a participant information sheet, and consent form. They will have an opportunity to read the PIS and consent form for at least 24 hours prior to attending the simulation event, where a researcher will answer any questions before inviting them to complete the consent form. Signed consent will be obtained from all participants. All participants are free to withdraw at any time from the study without giving reasons and without prejudice.

16b. Do you plan to include participants who are children (under 16 yrs)?
No

16c. Do you plan to include participants who are aged 16 or 17?
No

16d. Do you plan to include participants who lack capacity to consent?
No

16e. Do you plan to include any prisoners? No

16f. Are there special pressures that might make it difficult for people to refuse to take part in the study (e.g. subordinates)?
No. Junior doctors working under one of the study team may feel pressure to take part, however, this is not foreseen to be a significant risk as this potential conflict is managed on a regular basis within the Trust and College through established pathways. In addition, the NHS and its staff remain under pressure due to the ongoing demands of responding to the pandemic. Asking staff to devote further time to participating in this study may therefore create an additional burden. We will seek to address this by explaining the potential benefits of the technology - in reducing the stress/demands of caring for patients during the pandemic - in order for participants to feel they are directly contributing to direct improvements in the working lives of staff and care delivered to patients. In addition, we will ensure participants undertake the study at a manageable point during their standard working week, as is currently the case with all medical simulation/education, so the study does not take away important periods of rest and relaxation for participants to ensure that their physical and mental well-being is protected.

If any Junior Doctor feels pressure to take part, they will be encouraged to report this through the Medical Education Department or Freedom to Speak Up Guardian at the Trust.

17. Participant Involvement: Risks, Requirements and Benefits

17a. Describe potential hazards, risks or adverse effects that may be associated with the study?

The work will be performed within a simulated environment, where the risks to staff members are minimised. The major risk comes through the potential for COVID-19 transmission as described in section 11.

There is the possibility that a subject may feel uncomfortable or disorientated whilst wearing the HL2 device. If this occurs, then they will have the opportunity to stop the simulation and then either recover and carry on or withdraw from the study. Members of the simulation faculty and study team will be present throughout, and if there is any sign that subject are at risk of injury from becoming disorientated whilst wearing the device they will step in to stop the simulation and support the participant to prevent injury.

17b. Will pregnant or nursing mothers be included?

Yes, possibly. There is no increased risk to pregnant women or breast-feeding mothers, and we see no reason not to include them.

17c. Does your study involve invasive procedures such as blood taking, muscle biopsy or the administration of a medicinal product?

No

17d. If medical devices are to be used on any participant, do they comply with the requirements of the Medical Devices Directives?

No; HoloLens 2 is not classified as a Medical Device.

17e. List the locations or sites where the work will be done:
Clinical Simulation Laboratory, Imperial College London, St Mary's Hospital, Praed Street, London, W2 1NY

17f. Will group or individual interviews/questionnaires discuss any topics or issues that might be sensitive, embarrassing or upsetting?
No; simulated scenarios only.

17g. Is it possible that criminal or other disclosures requiring action, e.g. evidence of professional misconduct, could be made during the study?
No

17h. Describe any expected benefits to the research participant
No direct benefit to participants.

17i. Under what circumstances might a participant not continue with the study, or the study be terminated in part or as a whole?

Following wide-spread global commercial use of HoloLens across multiple sectors and industries there have been no recorded cases of harm coming to users.

If participants do not wish to complete the simulation, this will be facilitated, and no participants will be made to continue. If any participant wishes to withdraw, they will be able to stop and will not be coerced into continuing. Any identifiable data collected for a participant that withdraws from the study will be destroyed and not used for the purposes of analysis.

18. Financial Incentives, Expenses and Compensation

18a. Will travel expenses be given?.
No expenses or other payments will be provided to participants or study team members.

18b. Is any financial or other reward, apart from travel expenses, being offered to participants?
No

18c. Has payment of the Experimental Test Allowance been considered (JSP 752, chap 10 section 3)? Yes, and deemed to be not applicable.

18d. If this is a study in collaboration with a commercial *organisation*
No.

19. Confidentiality, Anonymity and Data Storage

19a. What steps will be taken to ensure confidentiality
HL2 devices utilise up to date security procedures and protocols. They are remotely configured using the Microsoft 365 Admin Centre in conjunction with Microsoft Intune, and as such adopt multi-factor authentication when joining the Remote Assist / Teams tenant that will deployed in this study. The use of strong passwords will be enforced, and on location the devices will connect to the local secure Wi-Fi network only after having been specifically white listed by MAC address. The network is typically secured using WPA2 with the use of Pre-Shared Key (PSK) authentication. The approach has been approved for use within secure NHS networks, and will be overseen and authorised by an appropriate responsible individual in the ICT department of each participating organisation.

Data from the original MR outputs will be uploaded securely to an instance of a UK/EU-hosted GDPR-compliant secure cloud storage service such as Microsoft Azure which is in use in both the NHS and UK Military. The storage, retention and audit of data will follow all local policies and procedures and will receive approval from the responsible data protection officer at Imperial College which will act as data controller for the purposes of this study.

The Chief Investigator will preserve the confidentiality of participants taking part in the study and is registered under the Data Protection Act.

19b. Give details of any anonymisation procedures to be used (if applicable)
Participants entered into the study will be given an anonymised code, and their personal details will not be stored. A single master list for the anonymised code will be kept securely by the CI.

19c. Who will have access to the records and resulting data?
The CI only.

The study may also be audited by a Quality Assurance representative of the RGIT (Research Governance and Integrity Team) at Imperial College London. All necessary data and documents will be made available for inspection. The study may be subject to inspection and audit by regulatory bodies to ensure adherence to GCP and the NHS Research Governance Framework for Health and Social Care.

19d. Where, and for how long, do you intend to store the Consent Forms and other records?

The original copy of the signed and dated informed consent form will be retained at the participating organisation and is subject to inspection by representatives of the Sponsor, or representatives from Regulatory Authorities. All data will be stored for a minimum of 10 years (or according to changes in regulatory requirements). Data generated by this work will be processed in accordance with the Data Protection Act 2018. Retention and analysis will be conducted with regard to all local policies relating to the collection, holding and disclosure of data relating to individuals. The Principal and Co-applicants will act as custodians of the data and be responsible for its security. The CI will ensure the continued storage of all relevant data and documentation, even if they leave the clinic/practice or retire before the end of the required storage period. Delegation will be documented in writing. Videos kept on the study hard drive within the department of surgery will be re-formatted and all copies of the videos will be destroyed on completion of the study.

19e: Have the Consent Form(s) and Participant information been reviewed and confirmed to be DPA 2018/GDPR compliant in accordance with organisational arrangements?

Yes.

20. Publication Policy

The outputs from this study will be submitted for peer-review publication and presentation at relevant international conferences. The study team will write an internal report summarising the key learnings from the study.

21. Supporting Documentation

The following appendices, where applicable, are included in this document (please indicate):

- ☒ **Research sponsors checklist**
- ☐ **Letter to general practitioners**
- ☐ **Letter to parents/guardians**
- ☐ **Letter of other research ethics committee opinion or other approvals**
- ☐ **Details of MHRA approval and/or correspondence (*if applicable*)**
- ☒ **Copy of email recruitment circular/poster/press advertisement**
- ☒ **Questionnaire/topic guide/interview questions**
- ☐ **Evidence of permission from organisation (e.g. hospital/university) where research is to be conducted**
- ☐ **List of acronyms**
- ☐ **CVs of named investigators**
- ☐ **CV(s) of supervisor(s)**
- ☐ **CV of Independent Medical Officer (*if applicable*)**

Please list any other documents that you are submitting to support this application:

Appendix 1 – COVID-19 Screening Questionnaire/Checklist

Covid-19 check list

Complete this checklist every time you visit the hospital, whether for an assessment, test or on the day of your procedure.

If you answer **YES** to any of the questions then please **stay at home** and contact the hospital to rearrange the appointment.

If you answer **NO** to all the questions then please attend your appointment as planned.

If you were shielding, make sure you let the clinic or department know.

Today or in the last 14 days…

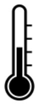	Do you have/have you had a fever (>37.8 degrees C)?	**Yes**	**No**
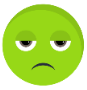	Do you have/have you had any of the following symptoms: cough, sore throat, loss of smell or taste, aches and pains, flu-like symptoms, diarrhoea?	**Yes**	**No**
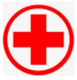	Have you tested positive for Covid-19?	**Yes**	**No**
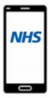	Have you been contacted by the NHS test and trace service and asked to self-isolate?	**Yes**	**No**
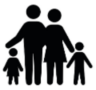	Has anyone in your household or your close contacts had or currently have: fever, cough, sore throat, loss of smell or taste, aches and pains, flu-like symptoms, diarrhoea?	**Yes**	**No**
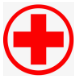	Has anyone in your household or your close contacts tested positive for Covid-19?	**Yes**	**No**
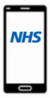	Has anyone in your household or your close contacts been contacted by the NHS test and trace service and asked to self-isolate?	**Yes**	**No**

Appendix 2 - example wording for recruitment e-mails / materials

Dear Colleagues,

COVID-19 has bought many challenges to how we can deliver the highest quality care to our patients whilst keeping our staff safe.

As you know, in the first wave of COVID-19 the Trust and Imperial College used the HoloLens device in a limited way to support the provision of remote care for our patients. This work showed that this new technology may help improve the quality of care we provide to patients and reduce the risk to staff from COVID-19.

The team who led this work has now been funded by the Ministry of Defence to further assess the effectiveness of the HoloLens headset, and to better understand how it can improve communication and teamwork, and reduce stress and risk for staff. This work is being undertaken in conjunction with the Medical Education Department and Simulation Team at St Mary's Hospital in support of our COVID-19 response locally.

We would like to invite you to volunteer to take part in a simulated trial of using the HoloLens device to help you assess and treat patients with COVID-19. The trial will take place over half a day in the simulation lab at St Mary's, and will involve teaching you how to use the technology, providing revision on the assessment and resuscitation of the critically unwell patient and undertaking two simulated patient scenarios.

If you would like more information, or would like to take part then please contact the simulation team - (email / telephone contact tbc).
