# Supplementary material for: Effect of Mixed Reality on Delivery of Emergency Medical Care in a Simulated Environment: A Pilot Randomized Crossover Trial
Source: JAMA Netw Open. 2023 Aug 28;6(8):e2330338. doi: 10.1001/jamanetworkopen.2023.30338 (PMC10463095; doi:10.1001/jamanetworkopen.2023.30338)
Supplement: Supplement 3. — Data Sharing Statement [file jamanetwopen-e2330338-s003.pdf]

Data Sharing Statement

Lawson. Effect of Mixed Reality on Delivery of Emergency Medical Care in a Simulated Environment: A Pilot Randomized Crossover Trial. *JAMA Netw Open*. Published online August 23, 2023. doi:10.1001/jamanetworkopen.2023.30338

Data
Data available: No

Additional Information
Explanation for why data not available: All relevant original data is available from the corresponding author on reasonable request.
